# The importance of timely cardiovascular risk screening in patients with premature ovarian insufficiency

**DOI:** 10.61622/rbgo/2026rbgoedt3

**Published:** 2026-07-17

**Authors:** Ana Carolina Japur de Sá Rosa e Silva, Maria Célia Mendes, Marcos Felipe Silva de Sá

**Affiliations:** 1 Universidade de São Paulo Faculdade de Medicina de Ribeirão Preto Department of Gynecology and Obstetrics Ribeirão Preto SP Brazil Department of Gynecology and Obstetrics, Faculdade de Medicina de Ribeirão Preto, Universidade de São Paulo, Ribeirão Preto, SP, Brazil

Premature ovarian insufficiency (POI) is associated with a significant increase in cardiovascular risk (CVR) throughout life. Early hypoestrogenism is the pathophysiological cornerstone of this process, since estradiol exerts essential cardioprotective effects, modulating lipid metabolism, endothelial function, insulin sensitivity, and the vascular inflammatory response. Epidemiological evidence confirms that early menopause and POI increase the incidence of coronary artery disease, stroke, and cardiovascular mortality, with a cumulative risk dependent on the duration of estrogen deprivation. The literature has several studies pointing to alterations in glucose metabolism, increased insulin resistance, dyslipidemia, increased blood pressure, metabolic syndrome, and other alterations in women with POI.^(1-4)^

The joint guideline of the Brazilian Society of Cardiology and the Brazilian Federation of Gynecology and Obstetrics Associations (FEBRASGO) highlights that hypoestrogenism negatively impacts the cardiometabolic profile and increases mortality, emphasizing that such risks are more linked to the menopausal transition than to chronological aging per se. The author draw attention to the increased risk of cardiovascular disease (CVD) in patients with POI.^(5)^ Some studies demonstrate early endothelial dysfunction, increased arterial stiffness, and greater carotid intima-media thickness, even at young ages and in the absence of traditional cardiovascular risk factors. The role of estrogens in triggering autoimmune mechanisms and cardiovascular inflammatory processes is also considered significant.^(6-8)^

Cardiovascular risk stratification in asymptomatic patients is particularly relevant within the context of primary health care. Patients at higher CVR should be identified in the population in order to initiate appropriate prevention strategies and, in some cases, to anticipate the start of screening. Young patients with POI should be included in this segment of the asymptomatic population to be investigated. However, systematic assessment of CVR in women under 40 years of age, especially in the absence of known risk factors, has not been recommended in the world literature, and medical society guidelines lack specific protocols for this age group.^(9-10)^

Information on the long-term cardiovascular consequences in these patients is still limited, as most data comes from women in physiological menopause, after 40 years of age. However, some author have incorporated this condition as a very important parameter in risk calculation. According to Kalantaridou et al.,^(11)^ women with POI present abnormal endothelial function assessed by flow-mediated dilation of the brachial artery, and it is possible to alter this condition with cyclic estrogen/progestogen hormone therapy.

However, because it is a relatively frequent disease, the use of protocols involving sophisticated techniques for screening incipient CVD, such as the measurement of carotid intima-media thickness, which are costly and require special equipment and human resources, would not be feasible in routine clinical practice. Even then, their implementation would not always allow for early identification of changes in these patients.^(1,12)^

Therefore, there is a clear need for simpler CVR screening methodologies in women with POI, specially by applying the already established routine markers at an earlier stage. Furthermore, traditional CVR calculators used to stratify risk categories are rarely applied to young women. Even in women over 40, these standard risk scores fail to account for the impact of hypoestrogenism.

Few studies have applied these risk assessment methodologies specifically to patients with POI. The author Gunning et al. (2020)^(1)^ used the 10-year CVD Framingham Risk Score (FRS) and the American Heart Association's suggested Cardiovascular Health Score (CHS) for risk calculation. They noted significant elevations in waist circumference, waist-to-hip ratio, blood pressure, and the prevalence of hypertension and metabolic syndrome compared to age-matched physiological menopausal controls. Notably, this was a retrospective study involving older patients with a mean age of 49.0 years (± 4.3) and a mean time since POI onset of 8.1 years (ranging from 6.8-9.6 years).^(1)^

In a recent study, Freaney et al. (2026), analyzing the incidence of myocardial infarction in a large population of 163,600 postmenopausal women aged 55-69 years, found that the risk of CVD was 1.41 (95% CI, 1.04-1.90) higher for Black women and 1.39 (95% CI, 1.03-1.87) higher for White women when associated with early menopause. According to the author "These results provide critical insights into premature onset of natural menopause as a risk-enhancing factor for coronary heart disease and underscore the importance of incorporating reproductive history in preventive efforts.^(13)^

It is therefore necessary to highlight the inadequacy of traditional scores applied to the female population after age 40 (natural menopause) and, in particular, to young women with hypoestrogenism, as is the case with POI. This reinforces the need for alternative approaches to lifetime risk assessment, thus considering these female-specific factors.

In 2019, the American College of Cardiology (ACC) and the American Heart Association (AHA)^(14)^ launched the Atherosclerotic Cardiovascular Disease ASCVD Risk Estimator Plus, which takes an important step by identifying that premature menopause (before 40-45 years) is independently associated with a 40% higher risk of atherosclerotic cardiovascular disease ("risk-enhancing factor"). According to the author, in women with early menopause, the ASCVD Risk Estimator Plus can be used to obtain baseline risk. If the risk is borderline or intermediate, the "early menopause" factor should be considered by the physician to intensify preventive measures, such as the use of statins, for example.^(14)^

Based on these studies demonstrating the impact of POI on cardiovascular risk at later ages, alongside the relevance of estrogen status and certain complications of the pregnancy-puerperal cycle (such as gestational diabetes and hypertension) to cardiovascular health, these conditions have more recently been designated as ‘reproductive risk markers’^(15,16)^ and incorporated into female-specific CVR stratification guidelines. Notably, these variables inform the clinical context when utilizing the AHA's new PREVENT™ Online Calculator "Clarity Prevention". This tool is available free of charge for CVR stratification calculation and can be applied to young patients starting at age 30.^(17)^

In conclusion, given the well-established evidence that hypoestrogenism significantly elevates CVD, it is vital that medical societies, particularly those managing endocrine and reproductive health, such as gynecology, endocrinology and pediatrics, decisively integrate CVR screening protocols for all women whose physical conditions involve estrogen deficiency, most notably patients with POI, irrespective of age.

## Data Availability

The research data are described in the article presented.
